# Supplementary material for: Protective Efficacy of Multiple Epitope-Based Vaccine against Hyalomma anatolicum, Vector of Theileria annulata and Crimean–Congo Hemorrhagic Fever Virus
Source: Vaccines (Basel). 2023 Apr 21;11(4):881. doi: 10.3390/vaccines11040881 (PMC10143353; doi:10.3390/vaccines11040881)
Supplement: Supplementary file 1 [file vaccines-11-00881-s001.zip › vaccines-2269057-supplementary.pdf]

Protective efficacy of multiple epitope-based vaccine against *Hyalomma anatolicum*, vector of *Theileria annulata* and Crimean-Congo haemorrhagic fever virus

Abhijit Nandi[1], Manisha[1], Vandana Solanki[2]; Vishvanath Tiwari[2], Basavaraj Sajjanar[3], Muthu Sankar[1], Mohini Saini[4], Sameer Shrivastava[3], S. K. Bhure[5] and Srikant Ghosh[*1]

Supplementary files-

Table S1. Primers for qRT-PCR (SYBR green):

Primer Code	5'--> 3'	F	Product Size (bp)	Accession Number
OC_INFG_F	ACCATGAGGTCATAAAGAAGAG	22	118	>D84216.1
OC_INFG_R	CATCCGAAATTCGAGTCAGA	20		
OC_IL2_F	CTGGAGGAAGTGCTTAACTT	20	120	>Z36904.1
OC_IL2_R	CACATGAATGTTTCAGATCCC	21		
OC_IL4_F	TCCTACCCGAAGTCATCAA	19	118	>AF169170.1
OC_IL4_R	CCTTGTGGTGGAGGTAGA	18		
OC_IL5_F	TACCAGAGTCTGCTGATAGG	20	104	>XM_002710201.3
OC_IL5_R	GTGTGTCTACACCTCGAAAG	20		

Table S2. Nature and sub-cellular localisation of the selected proteins

Target protein	SignalP 6.0	DeepLoc 1.0	Vaxijen 2.0	Allertop
TPM	Other	Golgi apparatus, Soluble	Antigen	Allergen
Fer2	Signal Peptide (Sec/SPI)<br>Cleavage site between pos. 15 and 16.<br>Probability 0.978782	Extracellular, Soluble	Non-Antigen	Non-Allergen
VgR	Signal Peptide (Sec/SPI)<br>Cleavage site between pos. 22 and 23.<br>Probability 0.979836	Cell membrane	Antigen	Allergen

Table S3. Predicted B-cell epitopes

Epitope position	Epitope sequence	Server used	Vaxijen 2.0
Fer2			
96	GVHVDMPPTATWMSV	Bepipder<br>IEDB	0.703

133	RLAADDDPQMADFLE	ABCpred	0.616
67	RFFRDQSSEEREHAQ	ABCpred<br><br>IEDB	0.944
2	FRIVVLALASAAWRD	ABCpred	1.196
111	DFLEQEFLAEQVKSID	ABCpred<br><br>IEDB	0.537
TPM			
77	DKALQAAEAEVAAHN	ABCpred<br><br>IEDB and Bepipred	0.672
110	TQKLEEASQAADESE	ABCpred<br><br>IEDB and Bepipred	0.93
43	VRSLQKKIQQIENEL	ABCpred<br><br>IEDB and Bepipred	0.519
193	LEEELRVVGNNLKSL	ABCpred<br><br>IEDB and Bepipred	1.01
VgR			
42	RCDGQNDCGNHKDT	Bepipred 2.0, ABCpred	1.79
93	DSSDEQDCHSSNCTG	Bepipred, ABCpred	1.79
138	GGVQNSSTTTPTPRC	Bepipred , IEDB	1.07
1027	TCDGHSDCSDSSDEK	Bepipred , IEDB	2.14
1067	RCDHDNDCEDSSDEV	Bepipred 2.0, ABCpred	1.47
594	DLNKVGAPVPVTLPV	Bepipred , IEDB	1.46
99	ADCHDSSDEQDCHSS	Bepipred 2.0, ABCpred	0.86
1743	NPAFNTRKTELLSED	Bepipred , IEDB	1
502	SNKRTGKQHHRVLRE	IEDB	1.77
1018	NGQCIPQDWTCDGHS	Bepipred , IEDB	0.99
1496	DGKDRKLVRGTGLSS	Bepipred 2.0	0.53
969	TCASHQYTCRGGVCL	Bepipred , IEDB	1.79

Table S4. Predicted CTL epitopes

Epitope position	Epitope sequence	Vaxijen 2.0	Allele (Bovine MHC-I)
Fer2			
0	MFRIVVLAL	1.325	BoLA-4:06301
10	SAAWAGNNL	1.148	BoLA-T7, BoLA-4:06301, BoLA-3:06602 BoLA-3:06601, BoLA-5:03901, BoLA-6:04001
14	AGNNLNEQV	1.478	BoLA-6:04001, BoLA-4:06301, BoLA-AW10
41	INLELHASL	1.68	BoLA-3:06801, BoLA-6:04001, BoLA-4:06301, BoLA-JSP.1, BoLA-AW10, BoLA-T7
75	QSSEEREHA	1.183	BoLA-T7, BoLA-6:04001, BoLA-4:06301
20	EQVNQNKYF	0.7252	BoLA-6:04001, BoLA-T7
54	MAAHLANNK	0.83	BoLA-6:04001, BoLA-4:06301, BoLA-T2a, BoLA-T7
41	INLELHASL	1.68	BoLA-JSP.1, BoLA-AW10,BoLA-T7, BoLA-3:06801, BoLA-6:04001, BoLA-4:06301
77	SEEREHAQK	1.48	BoLA-1:04201
137	LAADDDPQM	1.14	BoLA-T7, BoLA-4:06301, BoLA-6:04001
140	DDDPQMADF	0.89	BoLA-T7
141	DDPQMADFL	0.90	BoLA-6:04001, BoLA-T7
95	GTVSGVHVD	1.2	BoLA-4:06301, BoLA-T7
TPM			
5	KKKMQAMKL	0.831	BoLA-HD6, BoLA-D18.4, BoLA-T5, 1 BoLA-1:00902, BoLA-1:02301, BoLA-1:03101, BoLA-1:03102, BoLA-1:07401, BoLA-2:02201, BoLA-2:04701, BoLA-6:01301, BoLA-6:01302
26	EQQSREAAL	1.125	BoLA-HD6, BoLA-T2c, BoLA-T2b, BoLA-1:02101,BoLA-1:02901, BoLA-2:01601, BoLA-3:01001, BoLA-3:03801, BoLA-3:05002, BoLA-3:05001, BoLA-6:01301, BoLA-6:01302, BoLA-6:01401, BoLA-6:01402, BoLA-6:01502, BoLA-6:03401, BoLA-6:04101

86	AAHNRRIQL	1.084	BoLA-HD6, BoLA-JSP.1, BoLA-T2c, BoLA-T2a, BoLA-T7, BoLA-D18.4, BoLA-AW10, BoLA-1:02101, BoLA-1:02301, BoLA-1:02901,BoLA-1:02801, BoLA-1:04901, BoLA-1:06101, BoLA-1:06701, BoLA-2:00601, BoLA-2:00602, BoLA-2:01201, BoLA-2:01601, BoLA-2:01602, BoLA-2:01801, BoLA-2:01802, BoLA-2:02501, BoLA-2:02601, BoLA-2:02602, BoLA-2:02603, BoLA-2:03001, BoLA-2:04601, BoLA-2:04701, BoLA-2:04801, BoLA-2:05601, BoLA-2:05701, BoLA-2:06001, BoLA-2:06901, BoLA-2:07101, BoLA-3:00101, BoLA-3:00102, BoLA-3:00103, BoLA-3:00201, BoLA-3:00401, BoLA-3:00402, BoLA-3:00403, BoLA-3:01001, BoLA-3:01101, BoLA-3:01701, BoLA-3:01702, BoLA-3:01703, BoLA-3:02701, BoLA-3:02702, BoLA-3:03501, BoLA-3:03601, BoLA-3:03701, BoLA-3:03801, BoLA-3:05002, BoLA-3:05001, BoLA-3:05101, BoLA-3:05101, BoLA-3:05301, BoLA-3:05801, BoLA-3:05901, BoLA-3:06501, BoLA-3:06601, BoLA-3:06602, BoLA-3:06801, BoLA-3:07301, BoLA-4:06301, BoLA-5:03901, BoLA-5:07201, BoLA-6:01301, BoLA-6:01302, BoLA-6:01401, BoLA-6:01402, BoLA-6:01502, BoLA-6:04001, BoLA-6:04101
87	AHNRRIQLL	1.168	BoLA-HD6, BoLA-JSP.1, BoLA-D18.4, BoLA-AW10, BoLA-T5, BoLA-1:00902, BoLA-1:02101, BoLA-1:02301, BoLA-1:02901, BoLA-1:04901, BoLA-1:07401, BoLA-2:00501, BoLA-2:00602, BoLA-2:01601, BoLA-2:02501, BoLA-2:02601, BoLA-2:02602, BoLA-2:02603, BoLA-2:04601, BoLA-2:04701, BoLA-2:04801, BoLA-2:06001, BoLA-2:06901, BoLA-3:00101, BoLA-3:00102, BoLA-3:00103, BoLA-3:00201, BoLA-3:01001, BoLA-3:01101, BoLA-3:01701, BoLA-3:01702, BoLA-3:01703, BoLA-3:02701, BoLA-3:02702, BoLA-3:03501, BoLA-3:03801, BoLA-3:05002, BoLA-3:05001, BoLA-3:05101, BoLA-3:06501, BoLA-4:02401, BoLA-4:02402, BoLA-6:01301, BoLA-6:01302, BoLA-6:01402, BoLA-6:01502, BoLA-6:03401, BoLA-6:04001, BoLA-6:04101
105	RLKIATQKL	1.163	BoLA-HD6, BoLA-JSP.1, BoLA-T2c, BoLA-T2b, BoLA-T7, BoLA-D18.4, BoLA-T5, BoLA-1:00901, BoLA-1:00902, BoLA-1:02101, BoLA-1:02301, BoLA-1:02901, BoLA-1:02801, BoLA-1:03101, BoLA-1:03102, BoLA-1:04901, BoLA-1:06101, BoLA-1:06701, BoLA-2:00601, BoLA-2:00801, BoLA-2:00802, BoLA-2:01601, BoLA-2:01602, BoLA-2:02601, BoLA-2:02602, BoLA-2:02603, BoLA-2:04701, BoLA-2:04801, BoLA-2:05701, BoLA-2:06001, BoLA-2:06901, BoLA-3:00201, BoLA-3:01101, BoLA-3:01701, BoLA-3:01703, BoLA-3:03501, BoLA-3:05101, BoLA-3:06501, BoLA-3:06601, BoLA-3:06602, BoLA-3:06801, BoLA-3:07301, BoLA-4:02401, BoLA-4:02402, BoLA-5:00301, BoLA-5:03901, BoLA-5:06401, BoLA-5:07201, BoLA-6:01301, BoLA-6:01302, BoLA-6:01402, BoLA-6:01502, BoLA-6:04001, BoLA-6:04101

140	RMDGLEGQL	0.791	BoLA-HD6, BoLA-JSP.1, BoLA-T2c, BoLA-T7, BoLA-D18.4, BoLA-AW10, BoLA-1:00901, BoLA-1:02101, BoLA-1:02301, BoLA-1:02801, BoLA-1:04901, BoLA-1:06101, BoLA-1:06701, BoLA-2:00801, BoLA-2:00802, BoLA-2:02601, BoLA-2:02602, BoLA-2:02603, BoLA-2:04801, BoLA-2:05401, BoLA-2:05601, BoLA-2:05701, BoLA-2:06201, BoLA-2:06901, BoLA-2:07001, BoLA-3:00101, BoLA-3:00102, BoLA-3:00103, BoLA-3:00201, BoLA-3:03501, BoLA-3:03601, BoLA-3:05101, BoLA-3:06501, BoLA-3:06601, BoLA-3:06602, BoLA-3:06801, BoLA-3:07301, BoLA-5:00301, BoLA-5:03901, BoLA-5:06401, BoLA-5:07201, BoLA-6:01301, BoLA-6:01302, BoLA-6:01402, BoLA-6:01502, BoLA-6:03401, BoLA-6:04001
196	ELRVVGNNL	1.244	BoLA-HD6, BoLA-T2c, BoLA-T7, BoLA-1:06101, BoLA-3:01001, BoLA-3:05001, BoLA-6:01301, BoLA-6:01302, BoLA-6:01402, BoLA-6:01502
146	GQLKEARTM	0.708	BoLA-HD6, BoLA-T2b, BoLA-D18.4, BoLA-T5, BoLA-1:00901, BoLA-1:00902, BoLA-1:01901, BoLA-1:02001, BoLA-1:02101, BoLA-1:02301, BoLA-1:02901, BoLA-1:02801, BoLA-1:03101,BoLA-1:03102, BoLA-1:04201, BoLA-1:04901, BoLA-1:06101, BoLA-1:07401, BoLA-2:02601, BoLA-2:02602, BoLA-2:02603, BoLA-2:04701, BoLA-2:04801, BoLA-2:05501, BoLA-2:06201, BoLA-2:06901, BoLA-3:07301, BoLA-4:02401, BoLA-4:02402, BoLA-6:01301, BoLA-6:01302, BoLA-6:01401, BoLA-6:01402, BoLA-6:01502, BoLA-6:03401
189	KIVELEEEL	0.562	BoLA-HD6, BoLA-JSP.1, BoLA-T2c, BoLA-T7, BoLA-AW10, BoLA-1:02801, BoLA-1:04901, BoLA-1:06101, BoLA-1:06701, BoLA-2:00501, BoLA-2:00801, BoLA-2:00802, BoLA-2:02601, BoLA-2:02602, BoLA-2:02603, BoLA-2:04401, BoLA-2:05401, BoLA-2:05601, BoLA-2:05701, BoLA-3:00101, BoLA-3:00102, BoLA-3:00103, BoLA-3:00201, BoLA-3:01702, BoLA-3:03601, BoLA-3:05101, BoLA-3:05801, BoLA-3:06501, BoLA-3:06601, BoLA-3:06602, BoLA-3:06801, BoLA-3:07301, BoLA-4:06301, BoLA-5:00301, BoLA-5:03901, BoLA-5:06401, BoLA-5:07201, BoLA-6:01301, BoLA-6:01302, BoLA-6:04001
266	KYKAISDEL	0.625	BoLA-HD6, BoLA-1:06701, BoLA-2:04601, BoLA-2:04701, BoLA-2:04801, BoLA-3:01101, BoLA-3:01701, BoLA-3:05101, BoLA-3:06501, BoLA-3:06601, BoLA-3:06602, BoLA-3:06801, BoLA-4:02401, BoLA-4:02402, BoLA-5:00301, BoLA-5:03901, BoLA-6:01301, BoLA-6:01302, BoLA-6:04001
262	QEKEKYKAI	0.87	BoLA-T2b, BoLA-1:01901, BoLA-1:02001, BoLA-1:02901, BoLA-1:07401, BoLA-6:01401, BoLA-6:01402, BoLA-6:03401, BoLA-6:04101

199	VVGNNLKSL	0.898	BoLA-HD6, BoLA-JSP.1, BoLA-T2c, BoLA-T7, BoLA-AW10, BoLA-1:06701, BoLA-2:00501, BoLA-2:00601, BoLA-2:00602, BoLA-2:00801, BoLA-2:01601, BoLA-2:01602, BoLA-2:02501, BoLA-2:03001, BoLA-2:05601, BoLA-2:05701, BoLA-2:06001, BoLA-3:00101, BoLA-3:00102, BoLA-3:00103, BoLA-3:00201, BoLA-3:00401, BoLA-3:00402, BoLA-3:00403, BoLA-3:01001, BoLA-3:01101, BoLA-3:01701, BoLA-3:01702, BoLA-3:01703, BoLA-3:02701, BoLA-3:02702, BoLA-3:03501, BoLA-3:03601, BoLA-3:05101, BoLA-3:05301, BoLA-3:05901, BoLA-3:06601, BoLA-3:06602, BoLA-3:06801, BoLA-3:07301, BoLA-4:06301, BoLA-5:03901, BoLA-5:06401, BoLA-5:07201, BoLA-6:01301, BoLA-6:01302, BoLA-6:01502
VgR			
1693	RNRDKLAAL	0.93	BoLA-D18.4
1663	KSTSSSTAL	0.85	BoLA-D18.4
1618	GQFCNPVCL	1.61	BoLA-D18.4
1514	ALFEDWLYW	1.91	BOLA-T2A, BoLA-D18.4
1499	RKLVRGTGL	0.744	BoLA-D18.4
1429	LMFWAVWHE	0.59	BoLA-D18.4
1317	SMHGHAQHL	1.4	BOLA-HD6, BOLA-T2b, BOLA-T2C, BoLA-D18.4
889	TSTCRPHEL	0.62	BOLA-JSP.1, BoLA-D18.4
769	QKVFTGTPF	1.96	BoLA-D18.4
727	GQNPRVLPL	0.60	BOLA-JSP.1, HD6, T2b, BoLA-D18.4
595	NKVGAPVPV	0.74	BoLA-D18.4
507	KQHHRVLRE	0.64	BOLA-HD6, BoLA-D18.4
1511	FSIALFEDW	1.69	BoLA-JSP.1
1373	IHKPYHIAV	0.51	BoLA-JSP.1
1507	LSSPFSIAL	0.71	BoLA-T2C, BoLA-JSP.1
1300	YGEPFLLYM	0.69	BoLA-JSP.1
1281	CTCADGYAL	0.80	BoLA-T2C, BoLA-JSP.1
1682	ALLVLGYVL	1.15	BoLA- T2B, BoLA T2C, BoLA-HD6
1678	ALCVALLVL	0.68	BoLA-HD6
1293	RRYCKVQYG	0.59	BoLA-HD6
853	NGGCSHTCL	0.92	BoLA-T2C
247	PGSYSCHCL	1.7	BoLA-T2C
126	DCADASDEL	0.86	BoLA-T2C
522	GVHVYHPVL	0.74	BoLA-T2C, BoLA-T2b

Table S5. Predicted HTL epitopes

Epitope position	Epitope sequence	Vaxijen 2.0	Allele (Human MHC-II)
Fer2			
1	MFRIVVLALASAAWA	0.978	HLA-DRB1*08:02 HLA-DRB1*01:01 HLA-DQA1*01:02/DQB1*06:02 HLA-DPA1*02:01/DPB1*14:01 HLA-DRB1*12:01. HLA-DQA1*05:01/DQB1*03:01 HLA-DRB1*09:01. HLA-DPA1*03:01/DPB1*04:02. HLA-DQA1*03:01/DQB1*03:02 HLA-DRB1*04:01
2	FRIVVLALASAAWAG	1.4	HLA-DRB1*08:02 HLA-DRB1*01:01 HLA-DQA1*05:01/DQB1*03:01 HLA-DQA1*01:02/DQB1*06:02 HLA-DPA1*02:01/DPB1*14:01 HLA-DQA1*03:01/DQB1*03:02 HLA-DRB1*04:01 HLA-DQA1*04:01/DQB1*04:02 HLA-DRB1*12:01
3	RIVVLALASAAWAGN	1.51	HLA-DRB1*01:01 HLA-DQA1*01:02/DQB1*06:02 HLA-DQA1*05:01/DQB1*03:01 HLA-DRB1*08:02. HLA-DRB1*09:01 HLA-DQA1*04:01/DQB1*04:02. HLA-DPA1*02:01/DPB1*14:01 HLA-DQA1*03:01/DQB1*03:02 HLA-DRB1*12:01 HLA-DRB1*04:01
4	IVVLALASAAWAGNN	1.165	HLA-DQA1*05:01/DQB1*03:01 HLA-DRB1*01:01 HLA-DRB1*08:02 HLA-DQA1*01:02/DQB1*06:02. HLA-DRB1*09:01 HLA-DQA1*04:01/DQB1*04:02
5	VVLALASAAWAGNNL	1.055	HLA-DQA1*01:02/DQB1*06:02 HLA-DQA1*04:01/DQB1*04:02 HLA-DQA1*05:01/DQB1*03:01 HLA-DRB1*09:01 HLA-DRB1*01:01
33	RCRVGLQEQINLELH	0.705	HLA-DQA1*05:01/DQB1*02:01
36	VGLQEQINLELHASL	0.8532	HLA-DRB4*01:01
42	INLELHASLVYMQMA	0.78	HLA-DRB1*12:01 HLA-DRB4*01:01 HLA-DPA1*02:01/DPB1*14:01
43	NLELHASLVYMQMAA	0.6	HLA-DPA1*02:01/DPB1*14:01 HLA-DQA1*01:02/DQB1*06:02
44	LELHASLVYMQMAAH	0.51	HLA-DPA1*02:01/DPB1*14:01 HLA-DRB4*01:01 HLA-DQA1*01:02/DQB1*06:02
48	ASLVYMQMAAHLANN	0.64	HLA-DPA1*02:01/DPB1*14:01 HLA-DRB1*01:01 HLA-DRB4*01:01 HLA-DRB1*09:01 HLA-DRB5*01:01 HLA-DRB1*15:01
53	MQMAAHLANNKVARG	0.562	HLA-DRB3*02:02
54	QMAAHLANNKVARGG	0.78	HLA-DRB3*02:02
55	MAAHLANNKVARGGF	0.76	HLA-DRB3*02:02
140	ADDDPQMADFLEQEF	0.988	HLA-DQA1*01:01/DQB1*05:01
141	DDDPQMADFLEQEFL	1.017	HLA-DQA1*01:01/DQB1*05:01

142	DDPQMADFLEQEFLA	0.994	HLA-DQA1*01:01/DQB1*05:01 HLA-DPA1*01:03/DPB1*02:01
176	DTGLGEFLLDQQLRT		HLA-DRB3*01:01
TPM			
42	EVRSLQKKIQQIENE	0.553	HLA-DRB4*01:01, HLA-DRB1*11:01
43	VRSLQKKIQQIENEL	0.519	HLA-DRB4*01:01, HLA-DRB4*01:01
72	EEKDKALQAAEAEVA	0.827	HLA-DQA1*04:01/DQB1*04:02 1, HLA-DQA1*03:01/DQB1*03:02 1
73	EKDKALQAAEAEVAA	0.812	HLA-DQA1*03:01/DQB1*03:02 1, HLA-DQA1*04:01/DQB1*04:02 1, HLA-DQA1*05:01/DQB1*03:01
74	KDKALQAAEAEVAAH	0.982	HLA-DQA1*04:01/DQB1*04:02, HLA-DQA1*03:01/DQB1*03:02, HLA-DQA1*01:02/DQB1*06:02, HLA-DQA1*05:01/DQB1*03:01
75	DKALQAAEAEVAAHN	0.7	HLA-DQA1*05:01/DQB1*03:01, HLA-DQA1*03:01/DQB1*03:02, HLA-DQA1*04:01/DQB1*04:02, HLA-DQA1*01:02/DQB1*06:02
76	KALQAAEAEVAAHNR	0.74	HLA-DQA1*05:01/DQB1*03:01, HLA-DQA1*03:01/DQB1*03:02, HLA-DQA1*04:01/DQB1*04:02,
77	ALQAAEAEVAAHNRR	0.829	HLA-DQA1*05:01/DQB1*03:01
85	VAAHNRRIQLLEEDL	1.00	HLA-DQA1*05:01/DQB1*02:01
110	TQKLEEASQAADESE	0.94	HLA-DQA1*03:01/DQB1*03:02 1, HLA-DQA1*04:01/DQB1*04:02
193	LEEELRVVGNNLKSL	1.287	HLA-DRB5*01:01, HLA-DRB3*02:02
194	EEELRVVGNNLKSLE	1.122	HLA-DRB5*01:01, HLA-DRB3*02:02
195	EELRVVGNNLKSLEV	0.911	HLA-DRB3*02:02, HLA-DRB5*01:01
196	ELRVVGNNLKSLEVS	0.994	HLA-DRB3*02:02
219	ETYEMQIRQMTNRLQ	0.787	HLA-DRB4*01:01
222	EMQIRQMTNRLQEAE	0.923	HLA-DRB1*04:01
223	MQIRQMTNRLQEAEA	0.715	HLA-DRB1*04:01
VgR			
55	SNCTGFRCHNNECIP	1.07	HLA-DRB1*04:01
104	NCTGFRCHNNECIPA	1.24	HLA-DRB5*01:01, HLA-DRB3*02:02
105	CTGFRCHNNECIPAH	1.14	HLA-DRB5*01:01, HLA-DRB3*02:02
106	TGFRCHNNECIPAHW	1.38	HLA-DRB4*01:01
107	GFRCHNNECIPAHWR	1.45	HLA-DRB1*04:01
118	AHWRCDQTEDCADAS	1.33	HLA-DRB1*04:01
119	HWRCDQTEDCADASD	1.34	HLA-DQA1*03:01/DQB1*03:02 1, HLA-DQA1*04:01/DQB1*04:02
120	WRCDQTEDCADASDE	1.33	HLA-DRB1*12:01 HLA-DRB4*01:01 HLA-DPA1*02:01/DPB1*14:01

130	RIGYKLSANKHSCAV	0.55	HLA-DPA1*02:01/DPB1*14:01 HLA-DQA1*01:02/DQB1*06:02
982	CLPLYWRCDGSEDCP	1.1	HLA-DPA1*02:01/DPB1*14:01 HLA-DRB4*01:01 HLA-DQA1*01:02/DQB1*06:02
983	LPLYWRCDGSEDCPD	1.05	HLA-DPA1*02:01/DPB1*14:01 HLA-DRB1*01:01 HLA-DRB4*01:01 HLA-DRB1*09:01 HLA-DRB5*01:01 HLA-DRB1*15:01
986	YWRCDGSEDCPDGDD	1.33	HLA-DRB3*02:02
1301	YGEPFLLYMLPNQIR	0.77	HLA-DRB3*02:02
1302	GEPFLLYMLPNQIRS	0.64	HLA-DRB1*01:01 HLA-DQA1*01:02/DQB1*06:02 HLA-DQA1*05:01/DQB1*03:01 HLA-DRB1*08:02. HLA-DRB1*09:01 HLA-DQA1*04:01/DQB1*04:02. HLA-DPA1*02:01/DPB1*14:01 HLA-DQA1*03:01/DQB1*03:02 HLA-DRB1*12:01 HLA-DRB1*04:01
1303	EPFLLYMLPNQIRSF	0.59	HLA-DQA1*05:01/DQB1*03:01 HLA-DRB1*01:01 HLA-DRB1*08:02 HLA-DQA1*01:02/DQB1*06:02. HLA-DRB1*09:01 HLA-DQA1*04:01/DQB1*04:02
1304	PFLLYMLPNQIRSFS	0.65	HLA-DQA1*01:02/DQB1*06:02 HLA-DQA1*04:01/DQB1*04:02 HLA-DQA1*05:01/DQB1*03:01 HLA-DRB1*09:01 HLA-DRB1*01:01
1672	LASILVSALCVALLV	0.97	HLA-DQA1*05:01/DQB1*02:01
1663	ASILVSALCVALLVL	0.96	HLA-DRB4*01:01
1664	SILVSALCVALLVLG	1.01	HLA-DQA1*03:01/DQB1*03:02, HLA-DQA1*04:01/DQB1*04:02
1665	ILVSALCVALLVLGY	0.96	HLA-DQA1*05:01/DQB1*02:01

Table S6. GalaxyRefine: Comparative VT1 and VT2 refinement values

Model	GDT-HA	RMSD	MolProbity	Clash score	Poor rotamers	Rama favored
VT1						
Initial	1.0000	0.000	3.262	97.3	1.9	88.0
Refined Model	0.9448	0.417	2.497	13.1	3.8	93.3
VT2						
Initial	1.0000	0.000	1.510	2.4	0.0	92.0
Refined Model	0.8173	0.764	1.306	2.3	2.4	98.0

Table S7. Predicted antigenic and physiochemical properties of the constructs

Properties	VT1	VT2
Antigenicity (Vexijen 2.0)	0.46	1.0046
Allergenicity (AllerTop v. 2.0)	Non-Allergen	Non-Allergen
MolProbity Score	2.26	1.32
Clash Score	7.07	1.21
Highly Preferred Conformations	88%	98%
preferred conformations	8%	2%
Outter	4%	0%
Z-score	-2.23	-4.08
Molecular weight (Da)	8156.32	5266.98
Theoretical PI	4.97	4.61
The estimated half-life is	1 hours (mammalian reticulocytes, in vitro).<br>30 min (yeast, in vivo).<br>>10 hours (*Escherichia coli*, in vivo).	1 hours (mammalian reticulocytes, in vitro).<br>30 min (yeast, in vivo).<br>>10 hours (*Escherichia col*i, in vivo).
The instability index (II) is computed to be	27.18<br>This classifies the protein as stable.	35.46<br>This classifies the protein as stable.
Aliphatic index:	83.04	101.63
Grand average of hydropathicity (GRAVY)	-0.191	-0.257

Table S8. Haddock 2.4: protein-protein docking parameters of best docked complexes of VT1-TLR-9/TLR-2/TLR-4

Receptor	TLR-9	TLR-2	TLR-4
HADDOCK score	-43.1 +/- 27.8	-59.8 +/- 16.7	-28.1 +/- 16.9
Cluster size	6	5	4
RMSD from the overall lowest-energy structure	0.5 +/- 0.3	9.4 +/- 0.3	5.6 +/- 0.3
Van der Waals energy	-89.3 +/- 11.5	-57.1 +/- 6.2	-54.3 +/- 1.6
Electrostatic energy	-265.8 +/- 46.5	-319.1 +/- 51.2	-347.5 +/- 80.1
Desolvation energy	-37.3 +/- 5.7	3.0 +/- 3.1	7.5 +/- 1.6
Restraints violation energy	1366.4 +/- 127.8	581.5 +/- 83.8	881.6 +/- 76.7
Buried Surface Area	2992.5 +/- 170.9	2309.2 +/- 125.1	2056.7 +/- 91.0
Z-Score	-2.2	-2.0	-1.2

Table S9. Haddock 2.4: protein-protein docking parameters of best docked complexes of VT2-TLR-9/TLR-2/TLR-4

Receptor	TLR-9	TLR-2	TLR-4
HADDOCK score	-31.7 +/- 8.2	-32.3 +/- 8.0	-20.1 +/- 10.6
Cluster size	12	28	4
RMSD from the overall lowest-energy structure	0.5 +/- 0.3	8.9 +/- 0.2	10.8 +/- 0.1
Van der Waals energy	-70.6 +/- 6.8	-49.3 +/- 5.2	-48.5 +/- 7.6
Electrostatic energy	-478.5 +/- 40.1	-315.1 +/- 72.4	-278.2 +/- 74.5
Desolvation energy	-12.3 +/- 2.5	-3.2 +/- 1.7	-1.2 +/- 1.6
Restraints violation energy	1468.9 +/- 97.7	831.7 +/- 178.1	851.9 +/- 75.0
Buried Surface Area	2755.0 +/- 40.8	1952.0 +/- 153.2	2102.0 +/- 210.4
Z-Score	-2.3	-1.5	-1.4

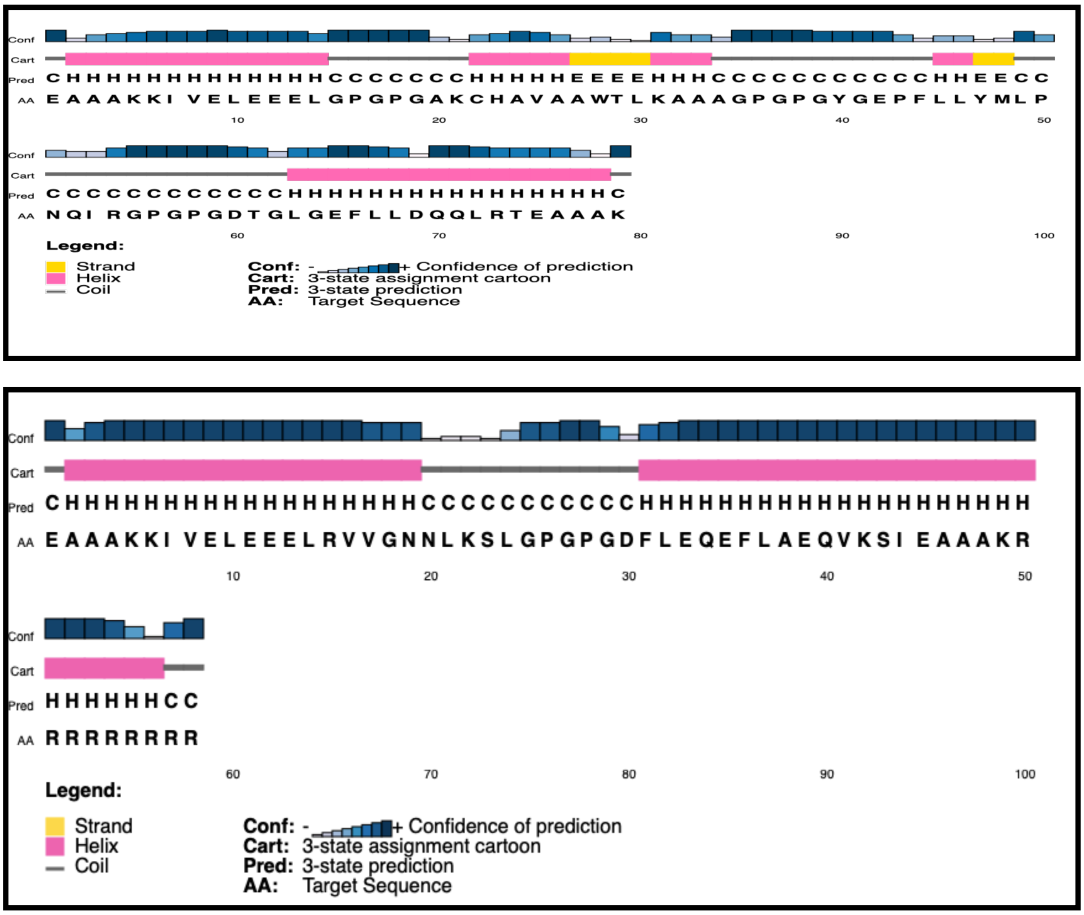

Figure S1. (A & B) Secondary structure prediction of VT1 (A) and VT2 (B) constructs using PSIPRED server. A, 49.4% alpha helices; 7.6% beta- strands; 43% coils. B, 75.86 alpha helices; 0% beta-strands; 24.13% coils.

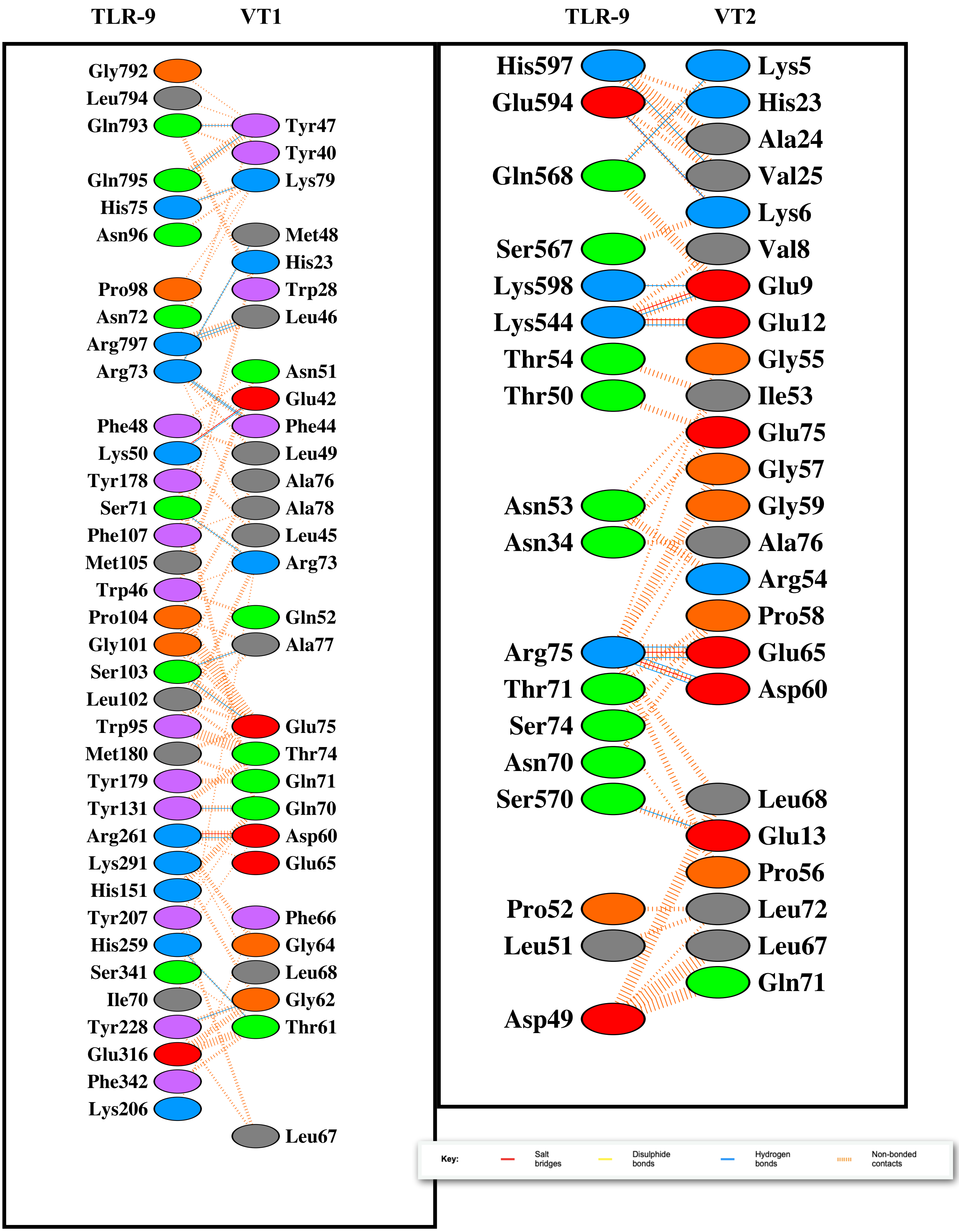

Figure S2. Schematic diagram of interactions between VT1 and VT2 with TLR-9 (Chain-A)

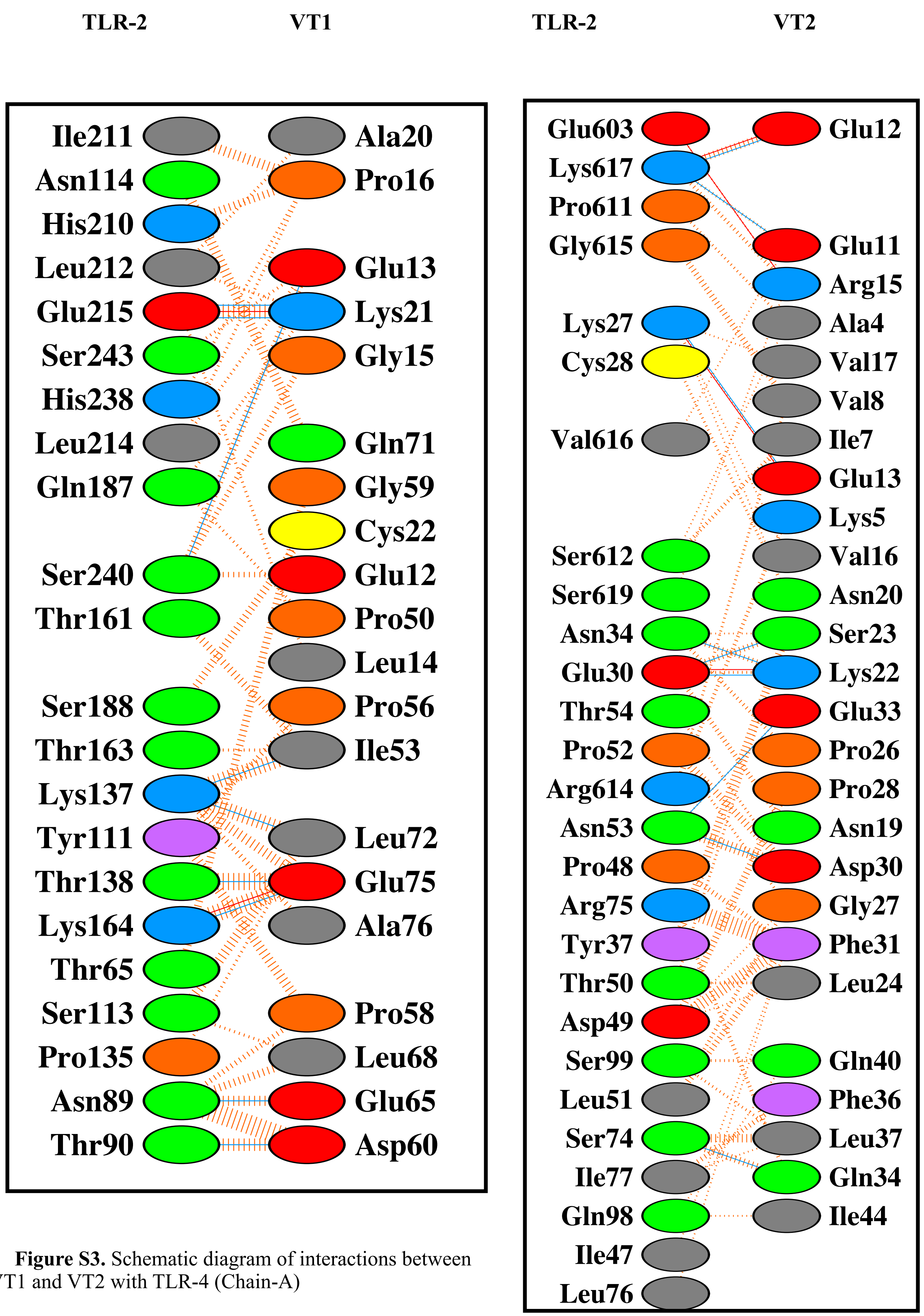

Figure S3. Schematic diagram of interactions between VT1 and VT2 with TLR-4 (Chain-A)

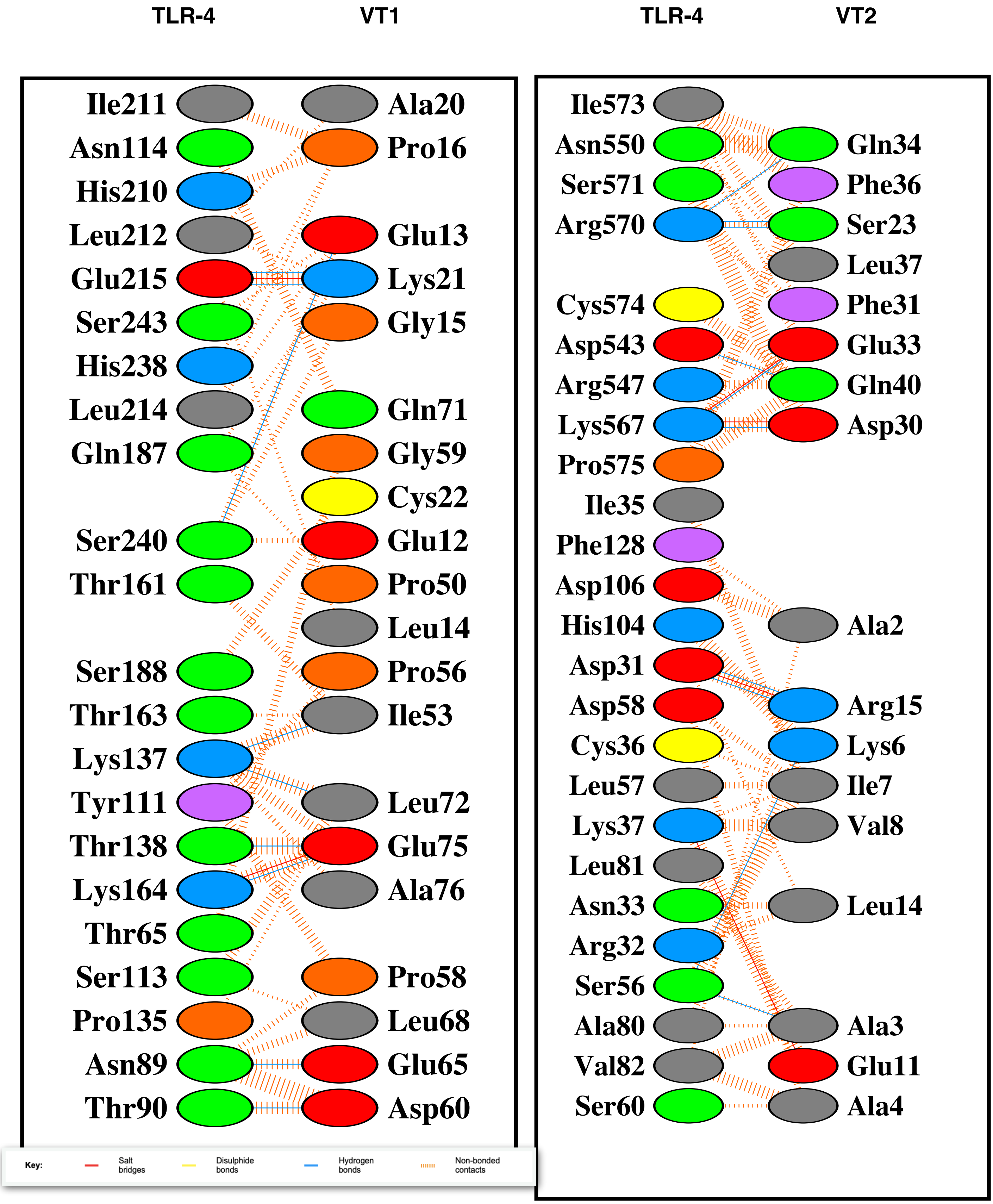

Figure S4. Schematic diagram of interactions between VT1 and VT2 with TLR-2 (Chain-A)

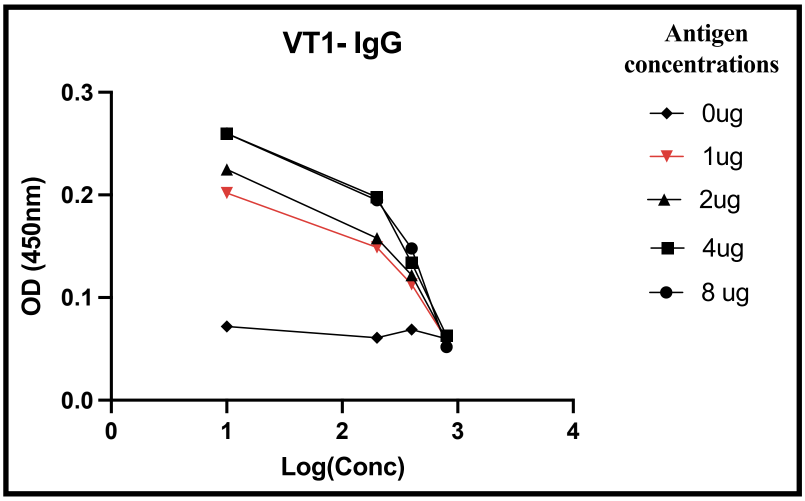

Figure S5. Checkerboard titration of VT1 antigen and IgG response

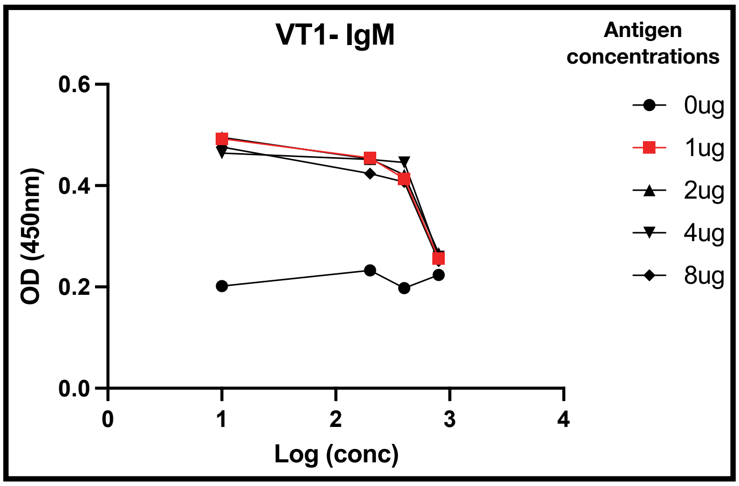

Figure S6. Checkerboard titration of VT1 antigen and IgM response

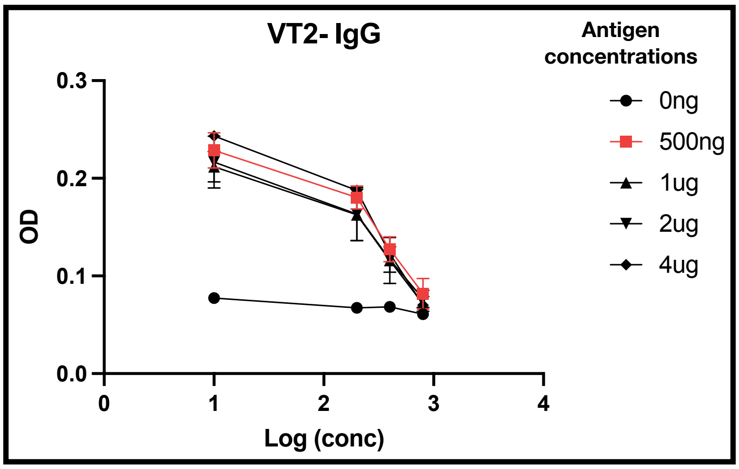

Figure S7. Checkerboard titration of VT2 antigen and IgG response

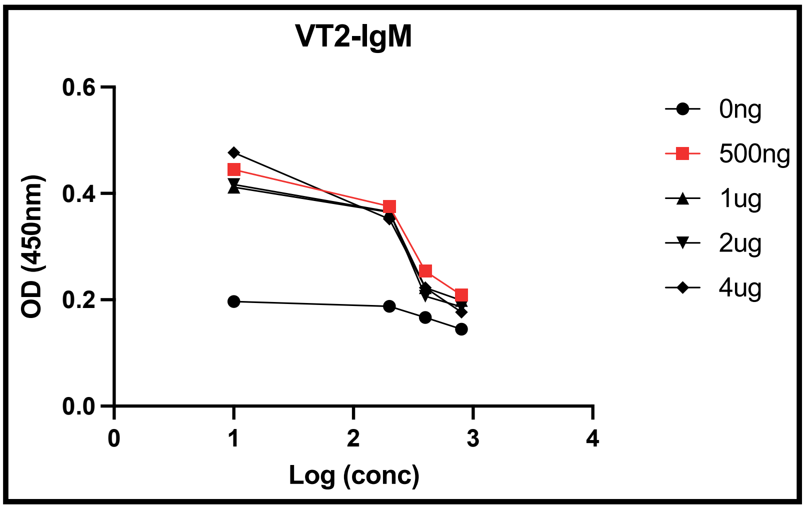

Figure S8. Checkerboard titration of VT1 antigen and IgM response

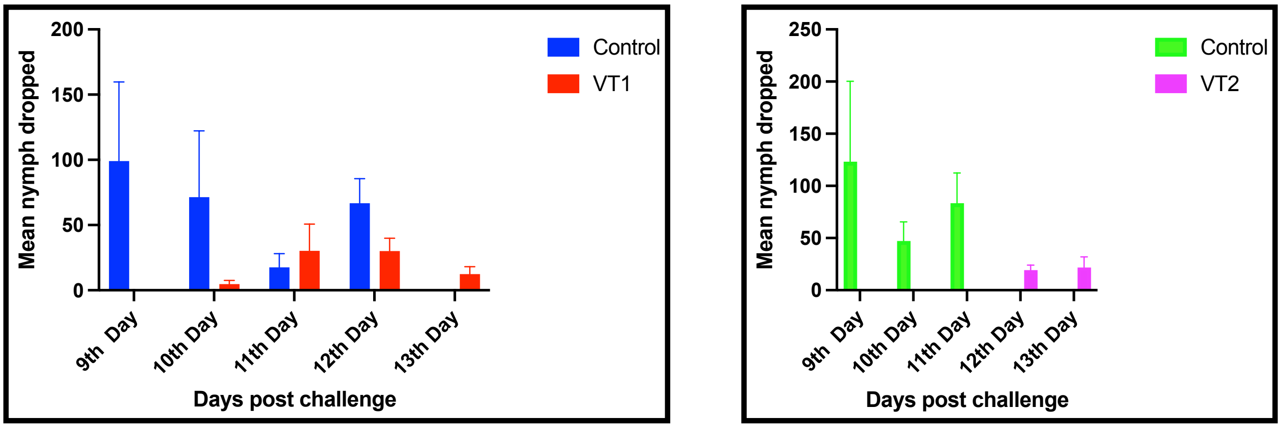

Figure S9. (A and B) Comparative feeding period of larvae fed on control and immunised group of rabbits. The bar represent mean number of nymphs dropped on a particular days of feeding from control and immunised animals and lines represent error values.

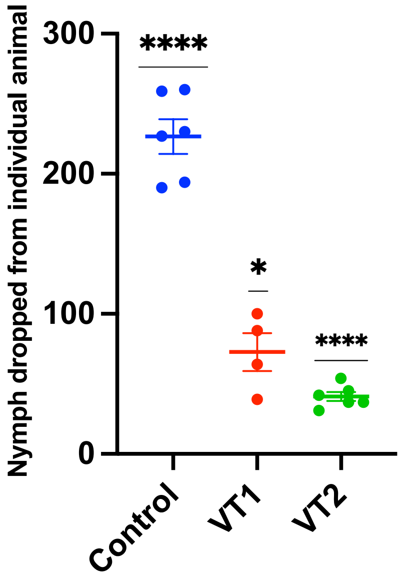

Figure S10. Showing comparative feeding pattern of larvae fed on each control and immunised group of rabbits.
